# Does a High Sugar High Fat Dietary Pattern Explain the Unequal Burden in Prevalence of Type 2 Diabetes in a Multi-Ethnic Population in The Netherlands? The HELIUS Study

**DOI:** 10.3390/nu10010092

**Published:** 2018-01-15

**Authors:** Merel J. Huisman, Sabita S. Soedamah-Muthu, Esther Vermeulen, Mirthe Muilwijk, Marieke B. Snijder, Mary N. Nicolaou, Irene G. M. van Valkengoed

**Affiliations:** 1 Division of Human Nutrition, Wageningen University, 6700 EV Wageningen, The Netherlands; m.j.huisman@amc.uva.nl (M.J.H.); S.S.Soedamah@uvt.nl (S.S.S.-M.); 2 Department of Public Health, Academic Medical Center, Amsterdam Public Health Research Institute, 1105 AZ Amsterdam, The Netherlands; e.vermeulen@amc.uva.nl (E.V.); m.muilwijk@amc.uva.nl (M.M.); m.b.snijder@amc.uva.nl (M.B.S.); m.nicolaou@amc.uva.nl (M.N.N.); 3 Center of Research on Psychology in Somatic Diseases (CORPS), Department of Medical and Clinical Psychology, Tilburg University, 5037 AB Tilburg, The Netherlands; 4 Biostatistics and Bioinformatics, Academic Medical Center, Department of Clinical Epidemiology, Amsterdam Public Health Research Institute, 1105 AZ Amsterdam, The Netherlands

**Keywords:** multi-ethnic, HSHF, T2D, western dietary pattern, HELIUS study

## Abstract

The risk for type 2 diabetes (T2D) in ethnic minorities in Europe is higher in comparison with their European host populations. The western dietary pattern, characterized by high amounts of sugar and saturated fat (HSHF dietary pattern), has been associated with a higher risk for T2D. Information on this association in minority populations is scarce. Therefore, we aimed to investigate the HSHF dietary pattern and its role in the unequal burden of T2D prevalence in a multi-ethnic population in The Netherlands. We included 4694 participants aged 18–70 years of Dutch, South-Asian Surinamese, African Surinamese, Turkish, and Moroccan origin from the HELIUS study. Dutch participants scored the highest on the HSHF dietary pattern, followed by the Turkish, Moroccan, African Surinamese, and South-Asian Surinamese participants. Prevalence ratios (PR) for T2D were then calculated using multivariate cox regression analyses, adjusted for sociodemographic, anthropometric, and lifestyle factors. Higher adherence to an HSHF diet was not significantly related to T2D prevalence in the total study sample (PR 1.04 high versus low adherence, 95% CI: 0.80–1.35). In line, adjustment for HSHF diet score did not explain the ethnic differences in T2D. For instance, the PR of the South-Asian Surinamese vs. Dutch changed from 2.76 (95% CI: 2.05–3.72) to 2.90 (95% CI: 2.11–3.98) after adjustment for HSHF. To conclude, a western dietary pattern high in sugar and saturated fat was not associated with T2D, and did not explain the unequal burden in prevalence of T2D across the ethnic groups.

## 1. Introduction

Diabetes is a major public health problem [1]. The risk for T2D in ethnic minorities in Europe is higher in comparison with their European host populations [2,3]. For example, a review of European studies published before April 2014 showed a higher prevalence of T2D in people of Middle Eastern and North African (2.7 times higher [2]), Sub-Saharan African (2.6 times higher [2]), and South-Asian (3.7 times higher [2]) origin in comparison with their European counterparts. Furthermore, a more recent study showed that the prevalence of T2D was up to 12 times higher in subgroups of African Surinamese, South-Asian Surinamese, Turkish, and Moroccan origin living in The Netherlands than among their Dutch counterparts [3]. African Surinamese, South-Asian Surinamese, Turkish, and Moroccans are the largest ethnic minorities living in The Netherlands; these groups form 22% of the total population in Amsterdam [4].

The cause of these observed ethnic differences in the prevalence of T2D is not fully understood. Differences in diet may partly underlie this unequal burden [5]. Studies show ethnic differences in diet in Europe [6]. For instance, ethnic minorities of Turkish, Moroccan, African, and South-Asian origin in The Netherlands consumed a higher amount of sugar sweetened beverages (SSBs), white rice, and processed and unprocessed meat in comparison with those of Dutch origin [6]. These single foods have been positively associated with risk for T2D in Europeans [7,8,9]. Only a few prospective studies have examined the association between single foods and the burden of T2D in populations of non-European origin. For instance, in native Hawaiians, an association was found between meat consumption and an increased risk for T2D [10]. In African American women, SSB intake was associated with an increased risk for T2D [11]. However, studies on the high risk in ethnic minorities in Europe are scarce.

Even fewer studies have investigated dietary patterns. Dietary patterns may better explain the interaction and combination of multiple nutrients and the risk for chronic diseases than single foods [12]. There is evidence that the western dietary pattern is associated with a higher risk for T2D [5,13]. The western dietary pattern is characterized by high intakes of sugar and fat, due to the high consumption of processed foods [14], SSBs, red and processed meat, sweets, refined grains, and fried foods, and low intakes of fruits and vegetables [11,13]. Most studies on western dietary patterns and T2D have been conducted within homogenous populations (European origin, predominantly ‘white’; [5,15]), with few studies in other populations [16,17,18,19]. A Hawaiian study suggested that a western dietary pattern contributed to a higher T2D risk in Japanese Americans and Native Hawaiians compared to Caucasians [18]. However, as diet and dietary patterns can vary by ethnicity [18], it is important to investigate the role of dietary patterns in relation to the burden of T2D in other ethnic groups.

The aim of this study is to investigate whether a western dietary pattern, characterized by high sugar high fat foods (HSHF), can explain the unequal burden of T2D in a multi-ethnic population in The Netherlands. To understand the contribution of individual food groups within the pattern, we will also determine the key elements of the pattern and look at their association with T2D. Finally, we will also combine these key elements into a simplified dietary pattern score and look at the association between this simplified dietary pattern score and T2D.

## 2. Materials and Methods

### 2.1. Study Design and Population

The current analysis is based on baseline data from the HELIUS (Healthy life in an Urban Setting) study, carried out by the Academic Medical Center Amsterdam and the Public Health Service of Amsterdam. The aims and design of the HELIUS study have been described before [20]. In brief, HELIUS is a large-scale prospective cohort study, which aims to unravel the causes of the unequal burden of disease among the largest ethnic groups in Amsterdam. Subjects were randomly sampled, stratified by ethnicity, from those aged 18–70 years listed in the Amsterdam Municipal Register [20]. Between 2011 and 2015, baseline data were collected by a questionnaire and a physical examination, and biological samples were obtained during the physical examination. The study protocols were approved by the AMC Ethical Review Board (approval code: NL32251.018.10), and all participants provided written informed consent.

For this present study, we used data from a sub-sample of men and women (18–70 years old) who participated in the physical examination and also completed an additional Food Frequency Questionnaire (FFQ) (*n* = 5053). Participants were excluded when data on T2D was missing (*n* = 34) and/or if participants had extreme energy intakes by using cut-off points according to Willet et al. ([21]; *n* = 325; women < 500 kcal/day or > 3500 kcal/day, men < 800 kcal/day or > 4000 kcal/day). We excluded 45 Dutch, 77 South-Asian Surinamese, 73 African Surinamese, 63 Turkish, and 68 Moroccan participants based on extreme energy intakes. This resulted in a total study population of 4694 participants: 1431 Dutch, 992 South-Asian Surinamese, 978 African-Surinamese, 586 Turkish, and 707 Moroccan origin participants.

### 2.2. Measures and Definitions

#### 2.2.1. The HSHF Dietary Pattern

We defined an HSHF dietary pattern, based on baseline dietary intake. A previous study showed that such an HSHF dietary pattern was associated with more depressive symptoms in this multi-ethnic population [22]. Due to the high co-morbidity between depression and T2D [23], we expected that this pattern might also contribute to ethnic differences in the burden of T2D. Habitual diet was measured using ethnic-specific Food Frequency Questionnaires (FFQs) that were specially developed for the HELIUS study [24] and validated against biomarkers (manuscript in preparation). Four questionnaires were developed, based on an existing Dutch FFQ; for Dutch, Surinamese, Turkish, and Moroccan ethnic groups. The questionnaires were comparable in terms of lay-out and consisted of similar and ethnic specific food items. We developed a single FFQ for the South-Asian Surinamese and African Surinamese participants because of similarities in commonly eaten foods [25].

#### 2.2.2. Reduced Rank Regression

The HSHF dietary pattern was derived using Reduced Rank Regression (RRR; [22]). RRR is a statistical method that derives latent variables (dietary patterns) from a set of predictor variables (food groups) that explain the maximum variation in another set of variables known as response variables (nutrients). RRR is an established method to derive dietary patterns and has been increasingly used in nutritional epidemiology [26]. Specifically, the HSHF dietary pattern used in this paper was originally derived in this population by Vermeulen et al. in a study of diet and depression [22]. RRR has some similarities with Principal Component Analysis (PCA) in that it is a reduction method that derives a latent variable. The advantage of RRR is that it accounts for total dietary intake of the study participants and the derived dietary pattern is closely related to the outcome measure due to the inclusion of intermediate response variables [22].

Previous studies showed that the intake of fat and sugar is associated with a higher risk of T2D, depression, and other metabolic diseases [27]. Therefore, as response variables, the nutrients mono- and disaccharides (g/day), saturated fatty acids (g/day), and total fatty acids (g/day) were used. As predictor variables, 51 food groups (g/day) were included based on nutrient profiles and culinary use.

In our population, heterogeneity is present due to differences in nutritional intake across ethnic groups, with ethnic minority populations consuming more high-sugar snacks and beverages but generally having a lower intake of saturated fat compared with the Dutch [6,28]. Moreover, our classification includes eight food groups based on ethnic-specific foods, which we have considered separately in order to reduce the heterogeneity within food groups. The explained variation in the response variables was 60% [22]. The RRR method assigns factor loadings to all food groups included in the analysis. Factor loadings indicate the strength of association between individual food groups and each of the derived latent variables (dietary patterns) and can be negatively or positively associated with the dietary pattern.

Individual participants receive a score for the derived dietary pattern based on their intake of all 51 food groups. Individual scores on the ‘dietary pattern’ are a reflection of the degree to which an individual’s dietary intake is consistent with that pattern. The degree to which individual food groups are representative of a dietary pattern is usually represented on the basis of cut-off points, which often vary from 0.15 (Miki et al. 2015 [29]) to 0.20 (Vermeulen et al. 2017 [22], Dekker et al. 2015 [30]), so we chose to report a cut-off point in between for the sake of interpretability. Therefore, food groups with factor loadings of ≥0.18 were regarded as being characteristic of the HSHF dietary pattern and were included as key elements of the HSHF dietary pattern in this study. However, as all 51 food groups receive a factor score, all food groups contribute to the respective dietary pattern. To give a complete overview of this, all factor loadings of the food groups included for RRR analyses are shown in Appendix A. In our study, we also derived a simplified pattern score, using the method described by Schulze et al. [31]. The simplified dietary pattern is the sum of unweighted standardized intakes of food groups with factor loadings ≥0.18 (Appendix A).

#### 2.2.3. Type 2 Diabetes

During the baseline physical examination, fasting blood samples were drawn. Whole blood was used to determine the concentration of HbA1c using HPLC technology (TOSOH, Tokyo, Japan). Plasma samples were used to determine the concentration of glucose by spectrophotometry, using hexokinase as the primary enzyme (Roche Diagnostics, Tokyo, Japan). Participants were asked to bring their prescribed medications, which were coded according to the Anatomical Therapeutic Chemical (ATC) classification [32]. Diabetes mellitus was considered to be present if the participants’ fasting glucose level was ≥7.0 mmol/L, if the HbA1c concentration was ≥6.5% (48 mmol/mol), if the participant was using glucose-lowering medication, or if the participant self-reported to have been diagnosed with diabetes by a health care professional [33].

#### 2.2.4. Ethnicity

Ethnicity was defined according to the country of birth of the participant, as well as that of their parents [34]. More specifically, a person was defined as of non-Dutch ethnic origin if he/she fulfilled one of two criteria: (1) he/she was born outside The Netherlands and had at least one parent born outside The Netherlands (first generation); or (2) he/she was born in The Netherlands but both parents were born outside The Netherlands (second generation). For the Dutch sample, we invited people who were born in The Netherlands and whose parents were born in The Netherlands. Participants of Surinamese ethnic origin were further classified according to self-reported ethnic origin (obtained by a questionnaire) into ‘African’, ‘South-Asian’, or ‘other’ [34]. In 1975, almost half of the population of the former Dutch colony Surinam migrated to The Netherlands. It is estimated that around 36% of these immigrants were South Asian Surinamese (descendants of indentured laborers, originally from the Indian subcontinent) and 41% African Surinamese (descendants of slaves, predominantly of African origin) [35].

#### 2.2.5. Covariates

Anthropometric measurements obtained during the physical examination were height, body weight, hip circumference, and waist circumference. These measurements were obtained in duplicate and the mean was used for analysis. Previous data showed that a larger waist circumference is associated with an increased risk of T2D and a larger hip circumference with a decreased risk of T2D [36]. These associations have been confirmed across ethnic groups [37,38]. Therefore, we adjusted for these variables separately. Due to the differences in body composition between ethnic groups, different cut off points for waist circumference were used for ethnic groups [37]. BMI (Body Mass Index) was calculated as weight/(height)^2^.

Other covariates were obtained by a questionnaire. Smoking was categorized as currently smoking or non-smoking. Alcohol use was reported as yes/no based on any consumption in the previous 12 months. We also adjusted for alcohol intake in grams/day and this did not change the association, so only the model with consumption in the previous 12 months is presented. Family history of T2D was categorized into three categories: yes/no/unknown. Physical activity was assessed with the Short Questionnaire to assess Health-Enhancing Physical Activity (SQUASH) and classified as meeting or not meeting the Dutch healthy Physical Activity (PA) norm of 30 min of moderate intensity PA daily [39]. Educational level was categorized into four categories: never been to school or elementary only (no school); lower vocational or lower secondary (lower education); intermediate vocational or intermediate/higher secondary (intermediate education); higher vocational or university (higher education).

#### 2.2.6. Statistical Analysis

Baseline characteristics for each ethnic group were reported as means for continuous variables, and percentages for categorical variables. Continuous variables that were not normally distributed, based on visual inspection, skewness, and kurtosis values, were described with a median and interquartile range (IQR). We also described differences in HSHF dietary pattern scores and single foods across ethnic groups.

The association of HSHF dietary pattern with T2D was determined by calculating prevalence ratios (PR) with multivariate cox regression analyses with the survival time set to 1, and adjusted for age, sex, and ethnicity. We estimated PRs instead of odds ratios (ORs) in this cross-sectional study, because ORs might overestimate the risk for T2D [40]. Cox regression may be a better alternative for the analysis of cross-sectional studies with binary outcomes than logistic regression, since the PR is more interpretable and easier to communicate to non-specialists than ORs [41].

The HSHF score was initially classified into quartiles. To retain power, we subsequently dichotomized the variable, to reflect low adherence (Q1 and Q2; ≤median in the full population) and higher adherence (Q3 and Q4; >median in the full population) of the pattern. In model 1, we included ethnicity and sex. The Likelihood Ratio test showed no evidence for effect modification for sex (*p* = 0.55) and for ethnicity (*p* = 0.59). In addition, none of the individual terms for the ethnic groups suggested a possible difference in the association (data not shown). Therefore, we continued our main analyses in the full population and only presented additional stratified results by ethnicity for the final models. We added educational level and family history to model 2; physical activity, smoking, and alcohol use to model 3; and waist circumference to model 4. We also additionally adjusted for BMI and hip circumference in separate models. Because these adjustments did not further change the association (data not shown), we only adjusted for waist circumference in the final model. Since energy intake lies in the pathway between dietary pattern and T2D, we adjusted for energy intake in model 5. We did not consider generation, as the number of second generation participants was limited.

The association of the key elements of the HSHF dietary pattern with T2D was determined by calculating the *z*-scores to standardize the food groups of each key element, and using multivariate cox regression to calculate the PRs per standard deviation change. Additionally, as an alternative to the HSHF dietary pattern score, we also analyzed the association of the simplified HSHF dietary pattern score (dichotomized into low and higher adherence) with T2D. Moreover, we hypothesized that being aware of T2D could influence the diet and thus affect the observed association. Therefore, we repeated the main analyses with only newly diagnosed T2D based on glucose/HbA1c levels, after the exclusion of all people with self-reported T2D and/or the use of anti-diabetic medication. Finally, we examined to what extent an HSHF dietary pattern explained ethnic differences in T2D.

The risk of T2D by ethnicity was estimated by multivariate Cox regression analysis with Dutch as the reference group. We adjusted the association of ethnicity with T2D for age, sex, educational level, family history, physical activity, smoking, alcohol use, waist circumference, and total energy intake. We then compared the PRs of this model with the PRs of the model that additionally included HSHF score. All statistical analyses were performed using IBM SPSS Statistics version 23.0 (IBM Corp., Armonk, NY, USA). A *p*-value < 0.05 was considered as statistically significant.

## 3. Results

The median age was around 50 years for the Dutch, South-Asian Surinamese, and African Surinamese participants, while the Turkish and Moroccan participants were approximately 10 years younger, reflecting a difference in population distribution in these two populations (Table 1) [3]. On average, 59.5% of participants were women. Educational level was lowest among Moroccan and Turkish participants and highest among the Dutch. The South-Asian Surinamese most frequently reported a family history of T2D. Lifestyle and anthropometry varied across ethnic groups. For instance, the total energy intake was lowest among South-Asian Surinamese participants and highest among the Dutch, and waist circumference was lowest among the Dutch and highest among Turkish participants. Finally, the prevalence of T2D was highest in South-Asian Surinamese (22.4%), followed by African Surinamese (15.6%), Moroccans (12.6%), Turkish (10.2%), and the Dutch (4.9%).

The Dutch scored highest on the HSHF dietary pattern, followed by the Turkish, Moroccan, African Surinamese, and South-Asian Surinamese (Table 2). This was also reflected in the higher adherence to the simplified dietary pattern for the Dutch compared to the other groups. The intake of the major food groups also differed by ethnicity. For instance, the Dutch had the highest intake of foods high in sugar and fat, like chocolates/sweets/cakes/cookies (mean of 30.8 (17.1–50.5) g/day), whereas the South-Asian Surinamese participants had the highest intake in savoury snacks (37.5 (16.2–65.5) g/day). Ethnic differences in intake were also observed for SSBs, high fat dairy products, red meat, pasta fast food, and potatoes.

After full adjustment, the association of the HSHF dietary pattern with T2D in the total population was not significant (Table 3, model 5). This was consistent for all ethnic groups (Appendix B). When participants who were already known to have T2D were excluded, the estimated association was stronger. A high score on the HSHF pattern (Q3 and Q4; >median in the full population) was non-significantly positively associated with a 1.33 (0.94–1.89) higher prevalence of newly diagnosed T2D in comparison with a low HSHF score (Table 3, model 4). After the addition of total energy intake in the final model, the estimated PR was reduced (PR 1.09 high versus low adherence, 95% CI: 0.65–1.82; model 5).

None of the food groups that highly contributed to the HSHF dietary pattern were independently associated with T2D prevalence (Table 4). The simplified pattern (the sum of these specific HSHF food groups) also showed no significant association with T2D; for instance, the PR of the high vs. low score was 0.85 (0.71–1.02) after adjusting for sociodemographic factors, anthropometric measurements, and lifestyle factors (model 4).

The higher prevalence of T2D among South-Asian Surinamese, African Surinamese, Moroccan, and Turkish participants in comparison with the Dutch was not explained by the HSHF dietary pattern (Table 5). For instance, the PR of the South-Asian Surinamese vs. Dutch changed from 2.76 (95% CI 2.05–3.72) to 2.90 (95% CI 2.11–3.98), after adjustment for the HSHF scores. These findings were similar in the analysis with newly diagnosed T2D only. For instance, the PR of the newly diagnosed South-Asian Surinamese vs. Dutch changed from 2.64 (95% CI 1.51–4.63) to 2.46 (95% CI 1.36–4.46).

## 4. Discussion

### 4.1. Key Findings

In line with previously reported results [3], we found large ethnic differences in the prevalence of T2D. Furthermore, our study found ethnic differences in the consumption of an HSHF dietary pattern, although we did not find an association between this dietary pattern and the prevalence of T2D. To conclude, our study found that an HSHF dietary pattern did not contribute to the ethnic differences in the prevalence of T2D in a multi-ethnic population in The Netherlands.

### 4.2. Discussion of Key Findings

As previously in part reported by Vermeulen et al. [22], we found ethnic differences in the HSHF dietary pattern. Our findings of ethnic differences in the HSHF dietary pattern score are also in line with previous studies in the US among other ethnic groups [19,42]. Akin et al. [42] previously reported that ethnicity is one of the most important factors associated with differences in dietary patterns in the US. Moreover, a study in Hawaii reported that Caucasians scored the highest on the western diet and Filipinos and Japanese scored the lowest (0.27 Caucasian vs. 0.16 Filipinos and −0.03 Japanese), respectively; [19]).

We did not find an association between the HSHF dietary pattern score and T2D prevalence. This suggests that the HSHF dietary pattern, while associated with depressive symptoms in our population [22], is not the best pattern to capture the dietary factors potentially associated with ethnic differences in T2D prevalence. This lack of association with T2D is also in line with the previous finding of a lack of association of patterns rich in fat and/or sugar with T2D in some non-European studies [17,18,19]. For example, a multi-ethnic study by Nettleton et al. [17] found no association between a ‘fats and processed meats’ pattern and T2D among white, black, Hispanic, and Chinese participants [17]. Another study, carried out in a multi-ethnic cohort in Hawaii, found no association between a western dietary pattern and the prevalence of T2D [19]. However, multiple prospective studies in European populations have found a positive association between a western/‘unhealthy’ diet and T2D prevalence [43,44,45]. Although we did not find a significant association, the estimated association was also the strongest for the Dutch host population in our study. We hypothesize that differences between studies may be related to subtle differences in the composition of the diet that underlies the western dietary pattern.

In addition, the lack of an association in our study may be related to the measurement of the HSHF dietary pattern in our study. The HSHF score was derived with RRR, which is considered as a powerful tool to derive dietary patterns [46]. However, other studies have used different methods, such as principal component analysis and cluster analysis [43,45]. The major strength of using RRR is that it is a posteriori method, which combines data from the study as food intake and prior evidence-based information for defining responses [46]. RRR makes use of all measured dietary variables to derive dietary patterns, as do other a posteriori methods. The advantage compared to a priori dietary patterns is that unexpected, population-specific foods can be identified in the derived dietary patterns. A disadvantage is that a high number of food groups makes the pattern more difficult to interpret as food groups with near zero loadings will contribute only a minor part to the overall HSHF score [26,31]. Previous studies by Schulze et al. [31] and Weikert et al. [26] suggested that a simplified pattern might enhance the relevance of the derived RRR dietary pattern. However, in line with our findings of the HSHF pattern, we did not find a positive association between the simplified pattern and T2D either. We could have considered different response variables. Commonly, response variables are disease specific intermediates (e.g., endogenous biomarkers or intermediate clinical phenotypes), whereas in our study, we used a combination of nutrients [31]. However, the response variables chosen in the current analyses have been previously associated with T2D and metabolic disease [27]. Furthermore, a previous analysis of the HELIUS study found that the HSHF dietary pattern was associated with more depressive symptoms [22], suggesting that the derived pattern is functioning.

We observed an important role for energy intake in the association between the HSHF dietary pattern and T2D in our study. Adjustment for energy intake greatly attenuated the observed association, implying that the role of an HSHF diet cannot be considered in isolation of energy intake. Thus, one may hypothesize that any potential effect of an HSHF diet, a common characteristic of a western diet, is attributable to the high energy density of the foods, as well as the likelihood of consuming them in excess, rather than just the nutritional composition of the foods themselves. Future research needs to further explore the role of energy intake in the association between diet and T2D.

Furthermore, unhealthy dietary patterns are often also characterized by a low intake of vegetables and fruit, which might not have been sufficiently accounted for by the HSHF pattern derived. Several studies showed that a high intake of fruit and vegetables reduces the risk of T2D [47]. It could therefore be possible that fruit and vegetable consumption confounded the association of HSHF and its components in our population. In a post hoc analysis, we verified whether this was the case by additionally adjusting for fruit and vegetable intake. However, the estimate did not substantially change the main association (data not shown).

An alternative explanation for the lack of association between HSHF diet and T2D is residual confounding by non-dietary factors. For instance, we were not able to explore the impact of early life determinants or individual changes in dietary patterns. Moreover, other unhealthy lifestyle factors, which may have been inadequately captured with the covariates we included in the models, might have a larger influence on the association with T2D than the HSHF pattern on its own. An unhealthy diet is generally associated with other unhealthy lifestyle factors. For instance, in the study of Fung et al. [48], participants who scored high on the western dietary pattern exercised less, watched more television, and were more likely to smoke. Another study showed that stress increased the risk of T2D [49]. In our study, we did not adjust for watching television and stress. Furthermore, the adjustment of some covariates may have been inadequate. For instance, we used the SQUASH to calculate physical activity; however, a study of the validity of the SQUASH showed that it is not an optimal basis for the comparison of physical activity between ethnic groups [39].

### 4.3. Strengths and Limitations

The strengths of our study were the large multi-ethnic sample sizes and the use of ethnic-specific FFQs [24]. A limitation was our cross-sectional study design, which meant that we were unable to investigate the longitudinal association between HSHF dietary pattern and incidence T2D, as was done in previous studies [5,15]. People with known diabetes may have modified their diet after their T2D diagnosis [50], which may have contributed to attenuation of the prevalence ratio towards one, thereby underestimating the associations. Exclusion of participants with known T2D strengthened the association towards a stronger effect estimate, but this association was still not statistically significant, perhaps due to a lack of power.

Another limitation might be the estimation of T2D used in our study. A single measurement of fasting plasma glucose and HbA1c was used to define T2D. Although our definition was based on the WHO criteria, we may have missed cases that would have been identified if an oral glucose tolerance test had been used [33].

To conclude, despite ethnic differences in a dietary pattern high in sugar and saturated fat, we found that this pattern was not associated with T2D and did not explain the unequal burden of T2D between ethnic groups. Thus, based on these findings, no recommendation can be made to contribute towards a reduction in (the ethnic differences in) the burden of T2D. Further longitudinal research on incident T2D in multi-ethnic populations is warranted to identify the factors underlying the observed unequal burden.

## Figures and Tables

**Table 1:** Baseline characteristics of the study population, by ethnicity.

*n* = 4694					
	Dutch (*n* = 1431)	South-Asian Surinamese (*n* = 992)	African Surinamese (*n* = 978)	Turkish (*n* = 586)	Moroccan (*n* = 707)
**Age (years) (median, [Q1, Q3])**	50.0 (38.0, 60.0)	49.0 (41.0, 57.0)	52.0 (44.0, 58.0)	43.0 (34.0, 50.0)	41.0 (32.0, 50.0)
**Women, *n* (%)**	798 (55.8)	589 (59.4)	658 (67.3)	311 (53.1)	439 (62.1)
Education level, *n* (%) *					
**1**	30 (2.1)	127 (12.8)	40 (4.1)	141 (24.1)	188 (26.7)
**2**	208 (14.6)	328 (33.2)	322 (33.1)	143 (24.4)	136 (19.3)
**3**	307 (21.5)	276 (27.9)	333 (34.2)	175 (29.9)	241 (34.2)
**4**	882 (61.8)	258 (26.1)	279 (28.6)	126 (21.5)	140 (19.9)
**Family history of T2D, *n* (%)**					
Yes	287 (20.1)	587 (59.2)	384 (39.3)	246 (42.0)	346 (48.9)
No	919 (64.2)	253 (25.5)	372 (38.0)	216 (36.9)	232 (32.8)
Unknown	225 (15.7)	152 (15.3)	222 (22.7)	124 (21.2)	232 (32.8)
Achieving norm for PA, *n* (%) **	1068 (74.6)	515 (51.0)	587 (60.1)	267 (45.6)	332 (47.0)
**Total energy intake (kcal/d), mean ± SD**	2171 ± 608	1967 ± 668	2040 ± 715	2144 ± 733	2050 ± 723
**Smoking, *n* (% yes)**	324 (22.7)	230 (23.2)	222 (22.8)	168 (28.7)	75 (10.6)
**Alcohol, *n* (% yes)**	1322 (92.4)	542 (54.9)	641 (65.7)	178 (30.5)	53 (7.5)
**BMI (kg/m^2^), mean ± SD**	24.8 ± 4.0	26.5 ± 4.8	28.1 ± 5.5	28.3 ± 5.1	27.6 ± 5.0
**Waist circumference (cm), mean ± SD**	89.5 ± 12.5	91.7 ± 12.6	93.5 ± 13.5	93.8 ± 12.8	92.8 ± 13.0
**Hip circumference (cm), mean ± SD**	100.7 ± 8.1	98.8 ± 9.2	103.9 ± 11.0	103.5 ± 11.0	104.1 ± 9.8
**Diabetes, *n* (% yes)**	70 (4.9)	222 (22.4)	153 (15.6)	60 (10.2)	89 (12.6)

* 1: Never been to school or elementary schooling only, 2: Lower vocational schooling or lower secondary schooling, 3: Intermediate vocational schooling or intermediate/higher secondary schooling, 4: Higher vocational schooling or university; ** achieving the Dutch norm of physical activity [39].

**Table 2:** HSHF dietary pattern and single foods by ethnicity.

	Dutch (*n* = 1431)	South-Asian Surinamese (*n* = 992)	African Surinamese (*n* = 978)	Turkish (*n* = 586)	Moroccan (*n* = 707)
**Mean HSHF score, mean ± SD**	0.20 ± 1.2	−0.57 ± 1.1	−0.41 ± 1.2	−0.08 ± 1.3	−0.26 ± 1.3
Dichotomous HSHF score, High adherence *, (%)	911 (63.7)	350 (35.5)	419 (42.8)	317 (54.1)	350 (49.5)
Single foods ** (g/day) (median, [Q1, Q3])					
**Sugar sweetened beverages**	14.3 (0.0, 80.5)	46.4 (7.1, 177.7)	64.3 (7.1, 244.6)	13.4 (0.0, 67.9)	17.9 (0.0, 93.5)
**Sugar/honey/jam**	8.6 (1.6, 21.8)	9.6 (2.4, 23.0)	10.7 (2.3, 24.8)	14.4 (4.3, 29.8)	15.0 (4.8, 32.0)
**Chocolates/sweets/cakes/cookies**	30.8 (17.1, 50.5)	15.5 (6.7, 30.1)	15.5 (5.5, 30.8)	20.8 (8.6, 42.2)	17.2 (6.9, 34.4)
**High fat dairy products**	46.0 (21.3, 94.3)	17.5 (3.3, 44.6)	18.6 (3.9, 50.3)	54.5 (24.1, 103.4)	64.3 (23.9, 142.9)
**Red meat**	37.1 (20.0, 58.0)	11.4 (1.5, 31.3)	22.5 (7.5, 45.7)	53.6 (28.6, 100.6)	40.2 (22.3, 75.9)
**Pasta**	34.3 (15.7, 68.6)	14.4 (0.0, 28.6)	15.2 (2.1, 28.6)	21.4 (9.8, 45.7)	24.3 (8.6, 45.7)
**Potatoes**	40.0 (20.0, 76.6)	14.6 (5.0, 35.9)	13.6 (2.6, 40.0)	20.2 (8.1, 40.0)	32.3 (12.5, 64.6)
**Fried potatoes**	6.9 (0.0, 13.9)	2.0 (0.0, 5.5)	5.4 (0.0, 15.2)	8.7 (1.7, 22.3)	8.9 (1.3, 25.8)
**Fast food**	13.4 (0.0, 33.5)	0.0 (0.0, 6.7)	0.0 (0.0, 3.3)	13.4 (0.0, 31.4)	6.5 (0.0, 21.0)
**Savoury snacks**	16.9 (7.5, 31.1)	37.5 (16.2, 65.5)	30.4 (12.2, 59.2)	10.9 (3.2, 24.9)	12.2 (3.9, 26.2)
**Mayonnaise and similar sauces**	3.5 (0.8, 7.5)	2.0 (0.0, 5.5)	2.9 (0.8, 6.6)	0.0 (0.0, 2.8)	0.2 (0.0, 4.3)
**Simplified HSHF dietary pattern score, mean ± SD**	0.82 ± 4.45	−1.38 ± 4.30	−0.78 ± 4.57	0.76 ± 5.26	0.74 ± 5.10
Dichotomous simplified HSHF dietary pattern score, high adherence *, (%)	844 (59.0)	366 (36.9)	404 (41.3)	327 (55.8)	406 (57.4)

* High adherence (Q3 and Q4; >median in the full population); ** Single foods chosen to represent food groups that were characteristic of the dietary pattern (factor loading ≥ 0.18).

**Table 3:** Association of high adherence to the HSHF dietary pattern with T2D in the total study population.

Cases	Model 1	Model 2	Model 3	Model 4	Model 5
	PR (95% CI)	PR (95% CI)	PR (95% CI)	PR (95% CI)	PR (95% CI)
**High vs. low score (total population)**					
94	1.01 (0.85, 1.21)	1.03 (0.86, 1.22)	1.03 (0.86, 1.22)	1.05 (0.88, 1.25)	1.04 (0.80, 1.35)
**High vs. low score (only newly diagnosed)**					
150	1.34 (0.95, 1.89)	1.34 (0.95, 1.88)	1.34 (0.94, 1.88)	1.33 (0.94, 1.89)	1.09 (0.65, 1.82)

Model 1: Adjusted for age, sex, and ethnicity; Model 2: Model 1 + adjusted for educational level and family history; Model 3: Model 2 + adjusted for physical activity, smoking, and alcohol use; Model 4: Model 3 + waist circumference; Model 5: Model 4 + total energy intake.

**Table 4:** Association between the simplified HSHF pattern score, individual key elements, and T2D.

	Model 3	Model 4	Model 5
	PR * (95% CI)	PR * (95% CI)	PR * (95% CI)
**Total population**			
**Simplified pattern score (high vs. low score)**	0.85 (0.71, 1.02)	0.85 (0.71, 1.02)	0.77 (0.62, 0.96)
**SSBs**	0.79 (0.70, 0.90)	0.81 (0.72, 0.92)	0.70 (0.70, 0.91)
**Sugar/honey/jam**	0.76 (0.68, 0.85)	0.81 (0.72, 0.90)	0.78 (0.70, 0.88)
**Chocolates/sweets/pastries**	0.83 (0.73, 0.94)	0.86 (0.76, 0.97)	0.83 (0.73, 0.94)
**High fat dairy products**	0.93 (0.85, 1.03)	0.97 (0.88, 1.06)	0.96 (0.87, 1.06)
**Red meat**	1.02 (0.93, 1.12)	0.98 (0.89, 1.08)	0.97 (0.88, 1.08)
**Pasta**	0.95 (0.84, 1.06)	0.95 (0.85, 1.07)	0.94 (0.84, 1.06)
**Potatoes**	1.00 (0.92, 1.08)	1.00 (0.91, 1.08)	0.99 (0.91, 1.08)
**Fried potatoes**	1.05 (0.97, 1.15)	1.05 (0.97, 1.15)	1.05 (0.96, 1.15)
**Fast foods**	0.93 (0.82, 1.06)	0.93 (0.82, 1.05)	0.92 (0.81, 1.05)
**Savoury snacks**	1.06 (0.99, 1.14)	1.05 (0.97, 1.13)	1.05 (0.97, 1.14)
**Mayonnaise and similar sauces**	1.03 (0.94, 1.13)	1.00 (0.91, 1.09)	1.00 (0.91, 1.09)
**Only newly diagnosed**			
**Simplified pattern score (high vs. low score)**	1.02 (0.72, 1.46)	1.06 (0.74, 1.51)	0.82 (0.53, 1.27)
**SSBs**	0.97 (0.80, 1.17)	0.99 (0.82, 1.19)	0.93 (0.76, 1.14)
**Sugar/honey/jam**	0.96 (0.81, 1.14)	1.03 (0.88, 1.20)	0.97 (0.82, 1.16)
**Chocolates/sweets/pastries**	0.88 (0.71, 1.10)	0.91 (0.74, 1.13)	0.82 (0.65, 1.04)
**High fat dairy products**	0.90 (0.74, 1.10)	0.97 (0.80, 1.17)	0.92 (0.74, 1.13)
**Red meat**	1.11 (0.95, 1.31)	1.06 (0.90, 1.26)	1.01 (0.84, 1.21)
**Pasta**	0.99 (0.80, 1.23)	0.98 (0.79, 1.21)	0.93 (0.74, 1.16)
**Potatoes**	0.95 (0.80, 1.12)	0.95 (0.79, 1.13)	0.90 (0.75, 1.09)
**Fried potatoes**	1.02 (0.85, 1.22)	1.03 (0.86, 1.23)	0.98 (0.81, 1.19)
**Fast foods**	1.09 (0.91, 1.31)	1.07 (0.89, 1.29)	1.04 (0.86, 1.26)
**Savoury snacks**	1.03 (0.89, 1.19)	1.01 (0.87, 1.17)	0.95 (0.81, 1.12)
**Mayonnaise and similar sauces**	1.11 (0.94, 1.30)	1.10 (0.93, 1.30)	1.05 (0.88, 1.26)

* = PR per SD change; Model 3: Adjusted for age, sex, ethnicity, education, family history, physical activity, smoking, and alcohol use; Model 4: Model 3 + waist circumference; Model 5: Model 4 + total energy intake.

**Table 5:** Ethnic differences in T2D, adjusted for known risk factors and HSHF score.

	Fully Adjusted Model *	Fully Adjusted Model * + HSHF Score
	PR (95% CI)	PR (95% CI)
**Total population: 594 cases**		
Dutch	1.00 (ref)	1.00 (ref)
South-Asian Surinamese	2.76 (2.05, 3.72)	2.90 (2.11, 3.98)
African Surinamese	2.17 (1.61, 2.93)	2.27 (1.66, 3.10)
Turkish	1.95 (1.31, 2.89)	2.01 (1.34, 3.00)
Moroccan	2.07 (1.42, 3.04)	2.13 (1.45, 3.14)
**Only newly diagnosed: 150 cases**		
Dutch	1.00 (ref)	1.00 (ref)
South-Asian Surinamese	2.64 (1.51, 4.63)	2.46 (1.36, 4.46)
African Surinamese	2.18 (1.29, 3.69)	2.04 (1.17, 3.56)
Turkish	2.03 (1.00, 4.10)	1.96 (0.96, 3.98)
Moroccan	1.25 (0.57, 2.71)	1.20 (0.55, 2.63)

* Fully adjusted model: Adjusted for age, sex, ethnicity, education, family history, physical activity, smoking, alcohol use, waist circumference + total energy intake.

## Appendix A

**Table A1:** Overview of all food groups included as predictor variables with their corresponding factor loadings derived by RRR in the HELIUS study.

Factor 1	Load
HSHF Dietary Pattern	
**Chocolates, sweets and pastries**	**0.29**
**Red meat**	**0.26**
**Sugar, honey and jam ***	**0.23**
**High-fat dairy products**	**0.23**
**Fried potatoes**	**0.2**
**Creamy sauces**	**0.2**
**Savory snacks ****	**0.19**
**Potatoes**	**0.19**
Sugar sweetened beverages *	0.18
Fast foods	0.18
Pasta	0.18
Nuts and seeds	0.18
Processed meat	0.17
Natural fruit juices	0.17
High-fiber bread products	0.17
Butter (spread and for cooking)	0.16
Low-fat dairy products	0.16
Chicken	0.16
Low-fiber bread products	0.15
Oil fat (not olive oil)	0.14
Eggs	0.13
High-fat margarine	0.13
Legumes	0.13
Soups	0.13
Other sauces	0.13
Coffee and tea	0.13
Olive oil	0.12
Peanut butter	0.12
Savory tomato sauces	0.11
Low-fat margarine	0.11
Vegetables	0.1
Rice and noodles	0.1
Organ meat	0.1
Fruit	0.1
Breakfast drinks	0.09
Lean fish and crustaceans	0.08
Alcoholic beverages	0.08
Fatty fish	0.08
Ayran	0.07
Olives	0.07
Borek and pogaca	0.07
Filled grape leaves	0.06
Roti	0.05
Pom	0.05
Light beverages	0.04
Vegetarian products	0.04
Avocado	0.03
Couscous	0.03
Moroccan pancakes	0.02
Soy dairy products	0.01
Water	−0.01

Food groups that were characteristic of the dietary pattern (≥0.18) and used in the simplified pattern are highlighted in bold; * SSB was restricted to soda’s, fruit juice, and sport drinks with sugar. We considered added sugar in beverages such as tea separately, and included this in the sugar/honey/jam category. ** Based on previous work (Vermeulen et al. 2017 [22]), we did not additionally distinguish between fast foods and savoury snacks in the RRR.

## Appendix B

**Table A2:** Stratified subgroup analyses for the association between HSHF dietary pattern score and T2D.

	Cases of T2D	Model 1	Model 2	Model 3	Model 4	Model 5
		PR (95% CI)	PR (95% CI)	PR (95% CI)	PR (95% CI)	PR (95% CI)
**Stratified subgroup analyses (total population)**						
Dutch (high vs. low score)	70	1.26 (0.74, 2.13)	1.33 (0.78, 2.26)	1.38 (0.81, 2.37)	1.28 (0.75, 2.17)	1.42 (0.71, 2.84)
SA Surinamese (high vs. low score)	222	1.20 (0.90, 1.60)	1.18 (0.88, 1.58)	1.15 (0.86, 1.54)	1.18 (0.88, 1.59)	1.07 (0.70, 1.62)
African Surinamese (high vs. low score)	153	0.91 (0.65, 1.29)	0.95 (0.68, 1.35)	0.94 (0.67, 1.34)	0.94 (0.66, 1.34)	0.91 (0.53, 1.56)
Turkish (high vs. low score)	60	0.95 (0.57, 1.60)	0.96 (0.57, 1.62)	0.96 (0.57, 1.63)	1.04 (0.61, 1.78)	1.03 (0.41, 2.58)
Moroccan (high vs. low score)	89	0.71 (0.45, 1.10)	0.70 (0.45, 1.09)	0.69 (0.44, 1.07)	0.70 (0.44, 1.09)	0.77 (0.39, 1.54)
**Stratified subgroup analyses (only newly diagnosed)**						
Dutch (high vs. low score)	24	1.79 (0.69, 4.62)	1.85 (0.71, 4.82)	1.86 (0.70, 4.91)	1.64 (0.62, 4.32)	2.48 (0.72, 8.57)
SA Surinamese (high vs. low score)	44	1.79 (0.96, 3.36)	1.72 (0.91, 3.23)	1.61 (0.85, 3.05)	1.66 (0.88, 3.16)	1.37 (0.55, 3.45)
African Surinamese (high vs. low score)	46	1.27 (0.69, 2.36)	1.20 (0.64, 2.24)	1.18 (0.63, 2.22)	1.11 (0.58, 2.12)	0.62 (0.23, 1.65)
Turkish (high vs. low score)	21	0.98 (0.41, 2.32)	0.95 (0.40, 2.27)	**	**	**
Moroccan (high vs. low score)	15	0.73 (0.25, 2.08)	0.64 (0.23, 1.83	0.60 (0.21, 1.74)	0.62 (0.21, 1.80)	0.52 (0.10, 2.79)

** = Sample size too small for correcting for all covariates in models 3–5. Model 1: Adjusted for age, sex, and ethnicity; Model 2: Model 1 + adjusted for education and family history; Model 3: Model 2 + adjusted for physical activity, smoking, and alcohol use; Model 4: Model 3 + waist circumference; Model 5: Model 4 + total energy intake.
